# Cord Plasma Concentrations of Visfatin, Adiponectin and Insulin in Healthy Term Neonates: Positive Correlation with Birthweight

**Published:** 2009-09

**Authors:** Ferhat Cekmez, Ozgur Pirgon, Asya Tanju, Osman Metin Ipcioglu

**Affiliations:** 1 *Department of Pediatrics, GATA Medical Faculty, Istanbul, Turkey*; 2 *Department of Biochemistry, GATA, Medical Faculty, Istanbul, Turkey*

**Keywords:** adiponectin, visfatin, insulin, birthweight

## Abstract

Objective: The aims of this study were to examine the relationships between adiponectin, insulin, visfatin and weight at birth in healthy term infants. Design and methods: Anthropometric parameters including weight, length were measured and plasma lipid profiles, insulin, visfatin and adiponectin concentrations in cord blood samples from 50 LGA and 50 AGA singleton infants born at term after uncomplicated pregnancies were assayed. Results: Mean visfatin and adiponectin levels were significantly higher in the LGA group than AGA group (11.8 ± 8 *vs.* 6.3 ± 5.5 ng/ml, *p*<0.001; 28.4 ± 3.9 *vs.* 25.7 ± 3.6 μg/ml, *p*=0.001; respectively). Insulin, triglyceride, total cholesterol, HDL-cholesterol and LDL-cholesterol levels did not differ significantly between LGA and AGA infants. Cord plasma adiponectin, visfatin and insulin levels correlated significantly and positively with birthweight (*p*=0.01, *p*<0.001, *p*<0.001; respectively) and with birthlength (*p=*0.01, *p*<0.001, *p=*0.01; respectively). Cord plasma adiponectin concentration correlated positively with visfatin level (*p=*0.005), but not with insulin level (*p*=0.8), and cord plasma visfatin concentration correlated positively with insulin level (*p*<0.001). Conclusion: High adiponectin and visfatin levels are present in the cord blood in LGA group. Cord plasma adiponectin and visfatin levels are positively correlated with birthweight. This suggests that adiponectin and visfatin may be involved in regulating fetal growth.

## INTRODUCTION

Adipose tissue, apart from storing fat, secretes a number of hormones called adipocytokines, two of which, visfatin and adiponectin, appear to play an important role in metabolism and energy homeostasis (3, 5). Adiponectin is exclusively expressed and secreted by the adipose tissue and is involved in glucose and lipid metabolism. Hypoadiponectinaemia has been shown to be associated with insulin resistance in animal and human studies (5). The primary mechanism by which adiponectin enhances insulin sensitivity appears to be through increased fatty acid oxidation and inhibition of hepatic glucose production (5). Plasma adiponectin levels are decreased in subjects with obesity and insulin resistance or type 2 diabetes mellitus, and are inversely correlated with visfatin and fasting insulin levels (7, 8, 10). Visfatin is a protein that is preferentially produced in visceral adipose tissue (2). It is expressed in the isolated subcutaneous adipose cells as well. Both tissue expression and plasma levels of visfatin increase in parallel with obesity. It has insulin-mimetic effects and lowers plasma glucose levels (3). Moreover, circulating visfatin concentrations were shown to increase in parallel with hyperglycemia (4). However, the data regarding pathophysiological role of visfatin in glucose homeostasis is limited. The role of adiponectin and visfatin in fetal growth has not been clearly determined. The aims of this study were to examine the relationships between cord blood adiponectin, insulin, visfatin levels and weight at birth in a large group of healthy term infants of normal, nondiabetic mothers.

## MATERIALS AND METHODS

### Subjects

In this study, 100 consecutive healthy infants of singleton pregnancies born at term (gestational age 37 weeks and higher), and whose stored cord plasma samples were available, were analysed between June 2006 and July 2007 in GATA Haydarpasa Military Hospital, Turkey. Gestational age at birth was calculated from the last menstrual period, and supported by ultrasound measurements. The Research Ethics Committee of Gulhane Military Academic Hospital approved the experimental protocol and written consent was obtained from the parents. Cord blood samples were collected into ethylenediamine tetraacetic acid (EDTA) tubes immediately after delivery and were stored at 4°C for a maximum of 12 h. They were then centrifuged, and the plasma was aliquoted and stored at −70°C until assayed. Routine measurements of birthweight and length were recorded and infants were divided into two groups using the Lubchenco intrauterine growth curves.

**Appropriate for gestational age (AGA) Group:** Normal birth weight, included 50 babies between 10th and 90th percentiles of Lubchenco intrauterine growth curve or birthweight between 2500 and 4000 grams.

**Large for gestational age (LGA) Group:** Large birth weight infant, included 50 babies of birthweight higher than 90th percentile or 4000 grams.

We prospectively enrolled the consecutive term AGA and LGA infants. Infants who were small for gestational age were regarded as high-risk newborns and were not included in this study. Additionally, infants of mothers with diabetes or any other medical complications during their pregnancy and infants with anomalies or who required intensive care were also excluded.

### Hormone and biochemical assays

Determination of visfatin levels was performed by enzyme immunoassay (visfatin C-terminal [human] EIA; Phoenix Pharmaceuticals, Belmont, CA). Minimum detectable concentration and intraassay and interassay coefficients of variation were 0.1 ng/mL and 5% and 12%, respectively. Adiponectin in cord plasma was assayed after 1:500 dilution using human adiponectin RIA kits (HADP-61HK; Linco Research), with interassay CV 6.9–9.3% and intra-assay CV 1.2–6.9%. All samples were analysed in duplicate. The concentrations of cord plasma total cholesterol, low density lipoprotein (LDL)-cholesterol, high density lipoprotein (HDL)-cholesterol and triglyceride were measured by using a Hitachi 7250 special autoanalyser (Hitachi, Tokyo, Japan). Cord plasma insulin concentration was measured by RIA using BioChem ImmunoSystems kits (BioChem Pharma Inc., Italy).

### Statistical analyses

Data were expressed as mean ± SD. Differences in the means of variables were tested using both parametric and non-parametric tests depending on the distribution of the variables. Correlation analyses were conducted using Spearman or Pearson correlation coefficients depending once again on the distribution of the variables. A probability value of less than 0.05 was considered significant. SPSS version 10.1 (SPSS, Chicago, IL) was used for analysis.

## RESULTS

Term infants were included in this study. LGA birthweight was 4.08 ± 0.23 kg, AGA birthweight was 3.3 ± 0.36 kg and LGA length was 52.9 ± 1.4 cm, AGA length was 49.5 ± 1.6 cm. Seventy-two per cent of study subjects were male. All anthropometric measurements, as expected, were significantly higher in LGA infants compared with AGA infants. The distribution of gender did not differ significantly between the LGA and AGA groups. Comparison of cord plasma concentrations of lipids and hormones between LGA and AGA infants were also shown in Table 1. Mean visfatin and adiponectin levels were significantly higher in the LGA group than AGA group (11.8 ± 8 *vs.*6.3 ± 5.5 ng/ml, *p*<0.001; 28.4 ± 3.9 *vs.* 25.7 ± 3.6 microg/ml, *p*=0.001). Insulin, triglyceride, total cholesterol, HDL-cholesterol and LDL-cholesterol levels did not differ significantly between LGA and AGA infants. Correlations between the cord plasma hormones and weight, lenght measurements of all the infants in the study were examined (Table 2). Cord plasma adiponectin, visfatin and insulin levels correlated significantly and positively with birthweight (*r*=0.2, *p*=0.01; *r*=0.33, *p*<0.001; *r*=0.21, *p*<0.001; respectively) and with birth length (*r*=0.18, *p*=0.01; *r*=0.22, *p*<0.001; *r*=0.18, *p*=0.01, respectively). Cord plasma adiponectin concentration correlated positively with visfatin level (*r=*0.13, *p*=0.005), but not with insulin level (*r*=0.05, *p*=0.8), and cord plasma visfatin concentration correlated positively with insulin level (*r*=0.2, *p*<0.001).

**Table 1:** Subject characteristics of total infants

Variable	LGA	AGA

Pregnancy outcome (*n*)	50	50
Female/male	15/35	13/37
Gestational age	39.9 ± 1.3^a^	38.5 ± 1.0
Birthweight (kg)	4.08 ± 0.23^a^	3.3 ± 0.36
Birth length (cm)	52.9 ± 1.4^a^	49.5 ± 1.6
Triglycerid (mmol/l)	0.28 ± 0.13	0.31 ± 0.27
Total cholesterol (mmol/l)	1.67 ± 0.48	1.69 ± 0.5
LDL-cholesterol (mmol/l)	0.5 ± 0.21	0.53 ± 0.27
HDL-cholesterol (mmol/l)	0.53 ± 0.2	0.55 ± 0.21
Insulin (pmol/l)	82.1 ± 56.2	68.4 ± 54.6
Visfatin (ng/ml)	11.8 ± 8^a^	6.3 ± 5.5
Adiponectin (μg/ml)	31.5 ± 5.9^a^	26.9 ± 5.6

Values are presented as mean ± SD.

a Data are given as means ± SD. difference at p<0.05 level.

**Table 2:** Pearson correlation coefficients between cord plasma hormones and weight and length at birth

	Birthweight	Birthlength

Adiponectin	0.2^b^	0.18^b^
Visfatin	0.33^a^	0.22^a^
Insulin	0.21^a^	0.18^b^

Adiponectin, visfatin and insulin were logarithmically transformed.

a *P*<0.001;

b *P*<0.01.

## DISCUSSION

This study clearly shows that cord plasma adiponectin, visfatin and insulin levels correlate significantly and positively with birthweight and lenght in the fetus. These results are in contrast to the previously reported that plasma adiponectin levels are decreased in subjects with obesity and insulin resistance and are inversely correlated with visfatin and fasting insulin levels (8, 10). The mechanism of the regulation of plasma adiponectin level and its negative association with body weight and adiposity is not yet fully understood. In some studies, adiponectin levels, in newborn infants, have been positively related with body weight, which is the converse of the association observed in adults (9). Tamakoshi *et al*., in a large study in adults, recently found a positive association between birthweight and adiponectin serum levels (11). Cianfarani *et al*., who examined adiponectin levels in SGA children, found low adiponectin levels closely related with birthweight (12). Conversely, Kim *et al*. were unable to demonstrate a relation between birthweight and serum adiponectin levels in adolescents (13). In animal studies, mice that were fed on a high-fat diet exhibited reduced adiponectin levels, while caloric restriction increased the levels of adiponectin (1). These results caused by chronic overfeeding on high fat diets, a common diet pattern in industrial societies with more than 50% of the total calories supplied from fat, rather than from weight loss or gain and that would explain the high adiponectin concentrations in cord blood found in this study (9).

This study found that cord plasma visfatin levels correlated significantly and positively with weight at birth, and with cord plasma insulin and adiponectin levels. Some studies showed that visfatin has been associated with intrauterine growth retardation, but the mechanism underlying effect of intrauterine growth remains unknown (6). Visfatin, a novel adipokine having insulin sensitizing properties additive to the effect of insulin, was reported to activate the insulin receptors in various cell types, increase glucose transport and lipogenesis in 3T3-L1 adipocytes or L6 myocytes, and decrease glucose production by hepatocytes *in vitro*. It not only acutely lowers the plasma glucose in mice but also improves insulin sensitivity and results in decreased glucose and insulin levels when administered to diabetic mice, an effect that is mediated by the insulin receptor itself with similar affinity but through binding a distinct site (2). Taken together, these findings suggest that visfatin could play a role in the association between obesity and diabetes mellitus, and because of correlation with high insulin levels in LGA group, LGA neonates should be examined closely for insulin resistance in future.

In conclusion, we show that high adiponectin and visfatin levels are present in the cord blood and significantly and positively correlated with weight and adiposity at birth that may be involved in some mechanisms that regulate fetal growth.
